# Presidential address: Quarantine guidelines to protect examinees from coronavirus disease 2019, clinical skills examination for dental licensing, and computer-based testing for medical, dental, and oriental medicine licensing

**DOI:** 10.3352/jeehp.2021.18.1

**Published:** 2021-01-19

**Authors:** Yoon-Seong Lee

**Affiliations:** President, Korea Health Personnel Licensing Examination Institute, Seoul, Korea; Hallym University, Korea


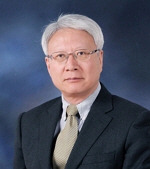


In 2020, many people experienced considerable suffering due to the coronavirus disease 2019 (COVID-19) pandemic. Health care workers worldwide are at the forefront of protecting people from this rapidly transmitted viral disease. Therefore, all agencies worldwide that administer licensing examinations for health professionals play a crucial role in ensuring a workforce that satisfies minimum requirements of competence. The Korea Health Personnel Licensing Examination Institute (KHPLEI) has continued to manage licensing examinations for health care professionals even in the COVID-19 pandemic situation, following the usual processes. It has prepared the following quarantine guidelines to ensure the safety of examinees during the examination process starting in fall 2020:

**Quarantine guidelines for examinees**
- If an examinee complains of fever, shivering, headache, muscle pain, laryngeal pain, rhinorrhea, nasal stiffness, dyspnea, vomiting, abdominal pain, diarrhea, or other symptoms, he or she will take the examination in a separate room.
- If an examinee is self-isolated as a suspected case of COVID-19, he or she will take the examination in a separate room.
- If an examinee is hospitalized due to COVID-19, he or she will take the examination in a separate room in the hospital or treatment center.
- For tests that include a lunch break, a lunch box and drinking water must be brought individually. The examinee should eat at his or her seat in the test room.
- When the examinee arrives at the test site in advance, he or she must follow social distancing regulations, maintaining a distance of more than 1.5 m between examinees.
- When entering the test site, the examinee should wear a mask, submit a self-checked sheet for any symptoms, check his or her body temperature, and disinfect his or her hands using hand sanitizer.

Thanks to examinees’ adherence to these quarantine guidelines, there were no cases of transmission of COVID-19 at any test sites from September 2020 to mid-January 2021.

On June 7, 2020, an agreement was reached with the Korean Institute of Medical Education and Evaluation (KIMEE) to make the *Journal of Educational Evaluation for Health Professions* (JEEHP) a joint official journal. The KHPLEI is pleased to have published articles on the history, development direction, and evaluation items of the KIMEE in 2020 [1]. The achievements of medical education in Korea have been largely based on the KIMEE, which launched medical school accreditation programs in 1998. The KIMEE and KHPLEI are the 2 most important institutions in the evaluation of medical education in Korea. If the research results produced by these 2 institutions are disseminated internationally through JEEHP, it will be beneficial to many countries’ licensing examination institutions and accreditation agencies for health professionals. We would like to encourage other license examination management institutions or accreditation agencies to make JEEHP an official journal to augment access to information and to exchange regional information internationally. Please contact the KHPLEI if you are interested.

Among the various topics discussed in my inaugural address in June 2019 [2], it is particularly noteworthy that a clinical skills examination for the Korean Dental Licensing Examination, which has been prepared for many years, will be conducted in 2021. The paper-and-pencil test of the Korean Medical Licensing Examination will be changed to a computer-based test (CBT) starting in 2022. Furthermore, it was officially announced that the Korean Dental Licensing Examination and Korean Oriental Medicine Licensing Examination will be conducted as a CBT starting in 2023. The first-degree Korean Emergency Medicine Technician Licensing Examination has been taken as a smart device-based test since 2017. The staff of KHPLEI have accumulated considerable technological and procedural experience, which can lead to the successful implementation of CBT.

Along with introducing CBT, we are preparing to implement the Korean Nursing Care Provider Licensing Examination as a year-round test using CBT starting in 2023. The establishment of year-round testing is an opportunity that can be applied to various licensing examinations. It will bring about a change in the number of examinations taken per year.

The modified Angoff method has been applied to the clinical skills examination for the KMLE. To establish a more reasonable standard setting for paper-and-pencil testing or CBT, researchers examined various methods, with a particular focus on the modified Angoff, modified Ebel, and Hofstee standard-setting methods [3].

The KHPLEI will continue to make efforts to advance licensing examinations and to facilitate test-taking this year. I would like to extend my gratitude for the editorial board members’ dedication to promoting JEEHP to an internationally top-tier journal. I also wish the submitters, reviewers, and readers of JEEHP a new year full of health and happiness even during the COVID-19 pandemic period.
